# Neural correlates of subjective cognitive decline in Alzheimer’s disease: a systematic review of structural and functional brain changes for early diagnosis and intervention

**DOI:** 10.3389/fnagi.2025.1549134

**Published:** 2025-04-23

**Authors:** Giulia Marafioti, Laura Culicetto, Desirèe Latella, Angela Marra, Angelo Quartarone, Viviana Lo Buono

**Affiliations:** IRCCS Centro Neurolesi “Bonino-Pulejo”, Messina, Italy

**Keywords:** Alzheimer’s disease, subjective cognitive decline, neuroimaging, machine learning, functional connectivity, early diagnosis

## Abstract

**Background:**

Subjective Cognitive Decline (SCD) is increasingly recognized as a preclinical stage of Alzheimer’s disease (AD), representing a critical window for early detection and intervention. Understanding the structural and functional neural changes in SCD can improve diagnosis, monitoring, and management of this early stage of disease.

**Methods:**

A systematic review was conducted using PubMed, Web of Science, and Scopus databases to identify studies examining neuroanatomical, neurofunctional, and neuroimaging findings in individuals with SCD. Inclusion criteria emphasized studies exploring SCD’s potential as an early biomarker for AD progression.

**Results:**

A total of 2.283 studies were screened, with 17 meeting the inclusion criteria. Evidence indicates that SCD is associated with cortical thinning and reductions in gray matter volume (GMV), particularly in the hippocampus, entorhinal cortex, and medial temporal lobe. Functional imaging studies reveal disruptions in the default mode network (DMN), executive control networks (ECN), and sensorimotor networks (SMN), indicating both compensatory mechanisms and early dysfunction. Dynamic functional connectivity studies report reduced brain activity efficiency, while graph theory analyses show decreased network integration. Advanced neuroimaging techniques and machine learning (ML) approaches demonstrate significant promise in detecting subtle neural changes in SCD, with applications for early diagnosis and monitoring disease progression.

**Conclusion:**

SCD represents a heterogeneous condition characterized by mixed compensatory and degenerative neural changes, marking a critical early stage in the AD continuum. Combining structural and functional brain alterations with advanced neuroimaging and ML methodologies provides valuable biomarkers for early detection. Future longitudinal and multimodal studies are essential to standardize methodologies, account for individual variability, and develop personalized interventions aimed at mitigating progression to dementia.

**Systematic Review Registration:**

https://www.crd.york.ac.uk/prospero/display_record.php?ID=CRD42024616052, CRD42024616052.

## 1 Introduction

Alzheimer’s disease (AD) is the most common cause of dementia and represents an irreversible neurodegenerative condition characterized by the progressive deterioration of cognitive functions (Livingston et al., 2017; Livingston et al., 2020). Currently, it is estimated that 6.5 million Americans aged 65 and older are affected by AD. In the absence of significant medical advancements to prevent, slow, or cure this disease, this number is projected to rise to 13.8 million by the year 2060 (Alzheimer's Association Report, 2024). Anatomically, AD is characterized by cerebral atrophy, primarily involving the temporal and parietal cortices, which leads to a reduction in the volume of regions crucial for memory and spatial orientation (Varghese et al., 2013). The hippocampus, a structure vital for long-term memory, is one of the earliest affected areas, and its degeneration contributes to initial memory impairment (Verfaillie et al., 2016). The hallmark pathological features of AD include the accumulation of extracellular beta-amyloid plaques between neurons and the presence of neurofibrillary tangles composed of tau protein within nerve cells (Chen and Mobley, 2019).

These pathological features impair intracellular transport systems and promote neurodegeneration (Kim et al., 2022). Consequently, there is a reduction in brain volume, with marked enlargement of cortical sulci and cerebral ventricles, reflecting the widespread loss of neural tissue that underlies the clinical symptoms of AD, including cognitive decline and difficulties in performing instrumental activities of daily living (Varghese et al., 2013; Kim et al., 2022). The progression of AD can be categorized into three distinct stages: the preclinical stage, during which pathogenic mechanisms are active but symptoms are not yet identifiable; the prodromal stage, characterized by the onset of mild cognitive impairment (MCI) that, while present, are not severe enough to meet the diagnostic criteria for dementia; and finally, the dementia stage (Dubois et al., 2007). One of the earliest signs of the disease could be identified in the subjective cognitive decline (SCD), which represents an initial stage marked by subtle cognitive changes the individual perceives (Rivas-Fernández et al., 2023).

SCD refers to persistent, self-reported cognitive decline, which may foreshadow the development of full-blown AD (Taya et al., 2018). Patients with SCD express concerns about their memory, attention, and other cognitive functions, even though their performance on standardized cognitive tests may still fall within the normal range (Jessen et al., 2014).

SCD represents a pre-symptomatic phase of significant interest. It allows for identifying early brain changes that occur before the onset of clinical symptoms. High spatial resolution neuroimaging techniques, such as structural magnetic resonance imaging (sMRI), enable in-depth, *in vivo* analysis of subtle brain structural changes in individuals with AD during the preclinical phase (Rivas-Fernández et al., 2023).

sMRI studies on SCD have revealed reduced volume in medial temporal lobe (MTL) structures, including the hippocampus and entorhinal cortex (Jessen et al., 2006; Striepens et al., 2010). Functional changes have also been observed in individuals with SCD. For instance, studies using task-based functional magnetic resonance imaging (fMRI) (Rodda et al., 2009; Hu et al., 2017) have found disruptions in neural networks during cognitive tasks in SCD subjects compared to healthy controls (HCs).

Early diagnosis of SCD related to AD is crucial for facilitating prevention and timely intervention in the clinical management of AD (Kim et al., 2022). In recent years, researchers have identified neuroimaging biomarkers capable of detecting early signs of neurodegeneration and changes in neurological disorders associated with AD, including the presence of SCD (Varghese et al., 2013). A previous sMRI study found that reduced cortical thickness, particularly in the temporal region, correlates with a more rapid memory decline among individuals with SCD (Verfaillie et al., 2016).

Recent studies indicate that individuals with SCD may exhibit abnormalities in AD biomarkers, such as reduced cerebrospinal fluid (CSF) Aβ42 levels and increased phosphorylated tau, suggesting a potential progression toward AD. Furthermore, the presence of such pathological biomarkers is associated with a higher risk of clinical progression to MCI or dementia compared to individuals with SCD without these biomarkers. Differences in cognitive performance and neuroimaging parameters between individuals with SCD with and without pathological biomarkers highlight the importance of these markers in the early identification of those at risk for clinical progression (Ulbl and Rakusa, 2023; Scarth et al., 2021).

In addition, it remains uncertain whether SCD represents a distinct condition or reflects a combination of factors including emotional states such as depression (Kleineidam et al., 2023), systematic disease, the use of specific drugs, and neurodegenerative processes (Monahan et al., 2024; Cheng et al., 2017). SCD frequently co-occurs with depression in older adults and is often a primary concern among those experiencing depressive symptoms. This strong association suggests that emotional factors, particularly depression, may significantly influence perceptions of cognitive decline (Jessen et al., 2014; Molinuevo et al., 2017; Burmester et al., 2016; Reid and MacLullich, 2006).

Currently, while no therapies can fully halt or reverse the progression of AD, recent advancements have led to the approval of disease-modifying treatments such as Lecanemab (approved by FDA and European Medicines Agency EMA) and Donanemab (approved by Food and Drug administration FDA, with EMA approval pending)(Jayaprakash and Elumalai, 2024; Martorana et al., 2025). Therefore, early diagnosis and implementation of interventions aimed at preserving cognitive abilities are crucial for managing and preventing further disease progression (1) Despite progress in understanding SCD, its heterogeneity and the variability of neuroimaging findings underscore the need for comprehensive studies to standardize diagnostic criteria and elucidate underlying mechanisms.

The aim of this review is to explore the structural and functional neural changes associated with SCD in individuals with AD. By identifying these specific neural correlates, this review seeks to enhance the accuracy of early diagnosis, improve the monitoring of neurodegenerative progression, and optimize clinical interventions in response to patients’ subjective report.

## 2 Materials and methods

**Figure 1:**
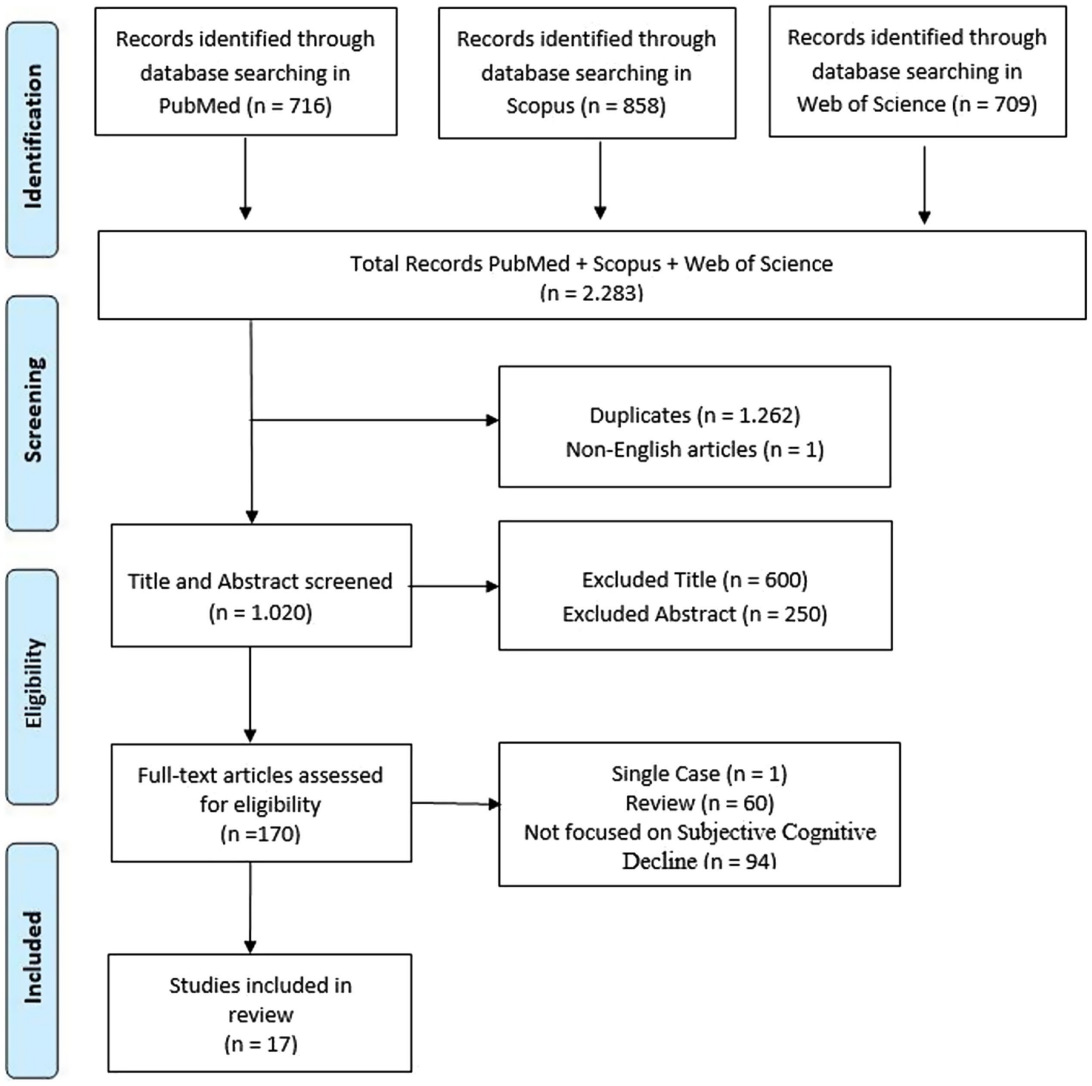
PRISMA flowchart showing identification, inclusion, and exclusion of studies in the systematic review.

This systematic review was conducted and reported in accordance with the Preferred Reporting Items for Systematic Review and Meta-Analyses (PRISMA) (see Figure 1) (Page et al., 2021). A protocol for this review was registered on PROSPERO under the registration number CRD42024616052.

### 2.1 PICO model

We employed the PICO (Population, Intervention, Comparison, and Outcome) model to shape our research question. Our target population comprises adults (>18 years) affected by SCD. The intervention involves the use of neuroimaging techniques (structural AND functional MRI,) and machine learning (ML) frameworks to detect and analyze structural and functional neural changes. For the comparison, we focused on HC subjects or individuals with MCI. The outcome is the identification of specific neural correlates, such as cortical thinning, changes in gray matter volume, functional connectivity alterations, and dynamic brain network properties correlated with SCD. This approach aims to enhance early diagnosis, improve the monitoring of neurodegenerative progression, and inform potential early intervention strategies.

### 2.2 Search strategy

The studies were identified by searching in PubMed, Web of Science and Scopus databases in July 2024. All the studies fulfilling our selected criteria were evaluated for possible inclusion. The search combined the following terms: (dementia OR “Alzheimer’s disease” OR “cognitive decline” OR “neurodegenerative disorders”) AND (“subjective cognitive impairment” OR “subjective cognitive decline” OR “subjective memory complaints”) AND (neuroimaging OR “brain imaging” OR MRI OR “magnetic resonance imaging” OR “diffusion tensor imaging” OR DTI).

The search terms were identified for the title and abstract. After removing duplicates, all articles were evaluated based on title and abstract. This research was not restricted by the year of publication for the articles considered.

The inclusion criteria were: (i) studies including patients with SCD; (ii) Studies using neuroimaging techniques (eg. sMRI and fMRI); (iii) articles in English language only.

Exclusion criteria were: (i) reviews and meta-analyses; (ii) duplicated studies; (iii) single case study; (iv) conference proceedings.

### 2.3 Study selection

To minimize bias and ensure a robust selection process, two authors (G.M. and L.C.) independently reviewed and extracted data from the studies. Any discrepancies were resolved through collaborative discussion and consultation with a third author (V.L.B). This multi-step approach guaranteed that at least three researchers independently assessed each article. In cases of persistent disagreement, all authors were involved in the final decision.

### 2.4 Data extraction and analysis

The studies that met the inclusion criteria were summarized based on the following points: (1) Study characteristics: type of study and the country in which the data had been collected; (2) Patients characteristics: the sample size, age, gender, duration of disease, and education; (3) Instruments utilized structural and functional MRI and ML and (4) main and relevant findings.

Following the full-text selection, data were extracted from the included studies and reported in a table using Microsoft Excel (Version 2021). The extracted data included: study title, first author name, year of publication, study aims and design, sample size, type of participants, type of intervention and control, baseline performance, type of outcome and time-points for assessment, results, and key conclusions.

Moreover, the agreement between the two reviewers (G.M. and L.C.) was assessed using the kappa statistic. The kappa score, with an accepted threshold for substantial agreement set at > 0.61, was interpreted to reflect substantial concordance between the reviewers. This criterion ensures a robust evaluation of the inter-rater reliability, emphasizing the achievement of a substantial level of agreement in the data extraction process.

### 2.5 Risk of bias within individual studies

**Figure 2:**
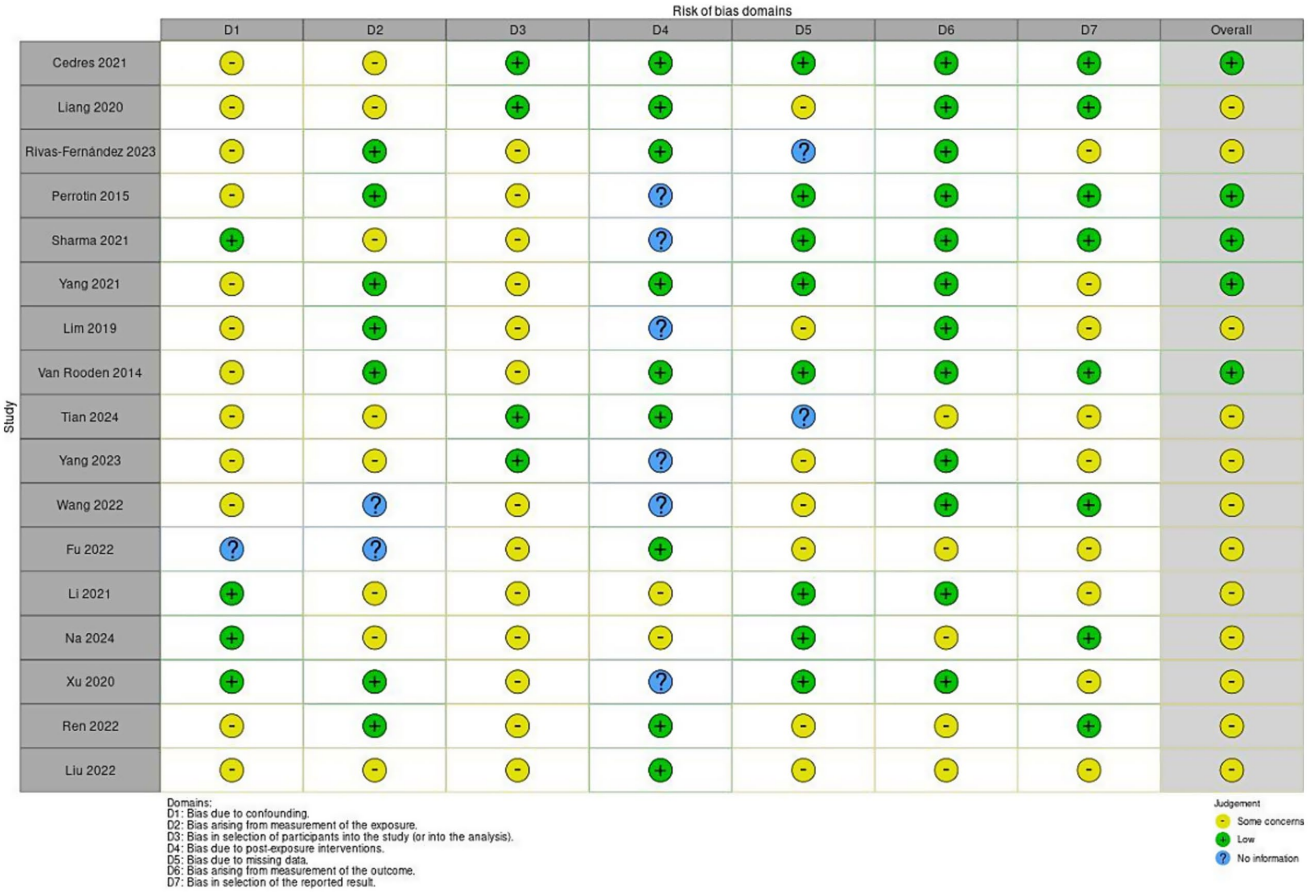
Risk of bias summary.

The risk of bias in the selected studies was independently assessed by G.M. and L.C. using the revised the Cochrane tool for non-randomised controlled studies-of exposures (ROBINS-E) tool (Figure 2), which comprises seven domains: (i) bias due to confounding, (ii) bias arising from measurement of the exposure, (iii) bias in selection of participants into the study (or into the analysis), (iv) bias due to post- exposure interventions, (v) bias due to missing data, (vi) bias arising from measurement of the outcome, (vii) bias in selection of reported result.

## 3 Results

### 3.1 Synthesis of evidence

A total of 2,283 articles were identified through database searches. Following the removal of duplicates, 1,020 studies were screened by title and abstract. Following full text selection, 17 studies were included for analysis. The selection process is shown in Figure 1.

### 3.2 Key findings from included studies

The literature reports various neuroanatomical, neurofunctional, and neuroimaging changes in individuals with SCD, providing a comprehensive overview of early brain alterations that may represent preclinical signs of MCI or AD (Table 1). Due to the limited number of studies included, a meta-analysis was not feasible; therefore, the synthesis is primarily qualitative.

**Table 1:** Main findings.

Study	Study Design	Sample size	Geographic Origin	Neuropsychological Assessment	MRI acquisition	Major findings
Rivas-Fernández et al. (2023)	Cross-sectional study	SDC group: 98 (73 female) mean age 68.12 ± 8.63	Spain, Santiago de Compostela (Western)	MMSE	T1-weighted 3D-MPRAGE sequence	The SCD group showed structural changes in frontal, parietal, and medial temporal regions which are key to AD.
				GDS-15		
				TMT-A		
				CAMCOG-R		
				CVLT		
		HC: 98 (73 female) mean age 65.80 ± 6.75		BNT		
				QSMC		
				IADL		

Fu et al. (2022)	Cross-sectional case–control observational study	SDC group: 35 mean age 64.54 ± 7.29	China, Beihang (Eastern)	MoCA	3.0 T Siemens system	Reduced global/local efficiency, clustering coefficients, and small-world properties in SCD with alterations primarily in the prefrontal, parietal, and subcortical regions. Age-related decreases in efficiency and nodal metrics were significant in SCD.
				AVLT		
				AVLT-I		
				AVLT-D		
		HC: 42 mean age 64.24 ± 6.16		AVLT-R		
				CDR		
				HAMD		
				ADL		
				SCD-I		
Wang et al. (2022)	Cross-sectional observational study	SDC group: 40 mean age 64.90 ± 8.31	China and Japan, Beijing, Kyoto (Eastern)	MMSE	3 T Siemens Magnetom Trio Tim MRI	Alterations in dynamic functional connectivity in individuals with SCD and AD, particularly within the sensorimotor and cognitive control networks, with changes in high-connectivity states correlating with cognitive performance,
				MoCA		
				AVLT		
				AVLT-I		
				AVLT-D		
				AVLT-R		
		AD group: 50 mean age 70.86 ± 9.87				
		HC: 60 mean age 62.57 ± 8.67				
Yang et al. (2023)	Cross-sectional observational study	HC: 60 mean age 64.64 ± 5.76	China, Guangxi, Shenzhen (Eastern)	MMSE	3.0-T MRI scanner	Morphometric changes in cortical and subcortical regions may lead to cognitive MCI, while compensatory mechanisms may help preserve cognitive function in SCD.
				MoCA		
				CDR		
		SDC group: 62 mean age 64.85 ± 5.62				
				GDepS		
				GDS		
		MCI group: 97 mean age 65.18 ± 6.56				
				AVLT		
				AVLT-R		
				AFT		
				BNT		
				STT-A		
				STT-B		
Tian et al. (2024)	Case–control observational study	SDC group: 30 mean age 62.20 ± 4.57	China, Beijing, (Eastern)	MMSE	3.0 T Siemens Prisma MR System	Different sub-regions of the insula exhibit varying connectivity patterns, associated with distinct functional brain networks such as the ECN, SN, and SMN, which are differently affected in patients with SCD.
				MoCA		
				ADL		
				AVLT		
		HC: 28 mean age 64.39 ± 4.76		CDR		
				CDT		
				HIS		
Liu et al. (2022)	Longitudinal observational study	SDC group: 22 mean age 74.4 ± 5.6	USA (North Carolina), China (Guangzhou, Shanghai), (Western + Eastern)	MMSE	3 T T1-weighted MRIs	The proposed JSRL method demonstrates enhanced performance in predicting SCD and MCI conversion.
		MCI group: 20 mean age 71.8 ± 2.9				
van Rooden et al. (2014)	Cross-sectional case–control observational study	HC: 27 mean age 68.9 ± 8.1	Netherlands, Leiden, Amsterdam, (Western)	MMSE	7-T MRI system	In subjects with SCI, an increased cortical phase shift detected through high-field MRI is associated with poorer memory performance. However, overall cortical phase shift in SCI subjects is not higher compared to HCs
				CAMCOG-R		
				WMS		
		SCI group: 18 mean age 65.5 ± 11.2				
				TMT-A		
				TMT-B		
		AD group: 28 mean age 71.2 ± 8.4				
				STROOP		
				GDS		
Lim et al. (2019)	Retrospective case–control observational study	pSCD group: 14 mean age 78.07 ± 3.65	South Korea, Seoul, (Eastern)	MMSE	1.5-Tesla MRI	The sSCD group exhibited widespread cortical atrophy in bilateral fronto-parieto-temporal regions, while the pSCD group showed more pronounced atrophy in AD-vulnerable areas, including the inferior parietotemporal and middle temporal regions.
				SNSB		
				GDS		
		sSCD group:21 mean age 70.71 ± 6.11				
		HC: 29 mean age 69.45 ± 5.30				
Yang et al. (2021)	Longitudinal observational study	HC: 82 mean age 73.88 ± 7.40	China, Suzhou, Tianjin, (Eastern)	MMSE	3.0 T MRI	SCD individuals exhibited lower dALFF and dReHo values but higher concordance in memory-related brain regions, including the hippocampus (HP), parahippocampal gyrus (PHG), and fusiform gyrus (FG), compared to MCI and HCs.
				MoCA		
				AVLT		
				WMS-LMII		
		SCD group: 94 mean age 71.07 ± 6.18		TMT-A		
				TMT-B		
		MCI group: 75 mean age 74.36 ± 8.42				
Sharma et al. (2021)	Cross-sectional case–control observational study	CU group: 26 mean age 71.42 ± 7.3	Canada, Toronto (Western)	MoCA	3 T	The increased risk of AD in patients with SCD may be explained by disrupted connectivity between the pDMN and the parahippocampal region.
				MFQ		
				GDS		
				CVLT-II		
		SCD group: 23 mean age 70.70 ± 5.5				
Perrotin et al. (2015)	Cross-sectional case–control observational study	SDC group: 17 mean age 69.12 ± 8.52	France, Caen, Lyon (Western)	MMSE	3- Tesla	Individuals with SCD exhibit hippocampal subfield changes similar to those seen in AD, underscoring the value of memory clinic-based SCD assessments in identifying early, pre-dementia stages of AD
				MADRS		
		AD group: 21 mean age 68.33 ± 9.48				
				STAIT-Trait		
				CDS		
		HC: 40 mean age 69.35 ± 6.37				
				RL-RI-16		
				BEM-144		
Liang et al. (2020)	Cross-sectional observational study	SDC group: 35 mean age 64.94 ± 5.955	China, Guangxi (Eastern)	MMSE	3.0 T MRI	Individuals with SCD exhibit structural and functional alterations in the hippocampus, which may serve as potential biomarkers for SCD.
				MoCA		
				CDR		
		HC: 32 mean age 63.03 ± 5.433		ADL		
				GDS		
				AVLT		
				BNT		
				TMT-A		
				TMT-B		
Cedres et al. (2021)	Cross-sectional observational study	SDC group: 225 mean age 54.64 ± 10.18	Sweden, Spain, USA, UK, (Western)	MMSE	3.0 T GE imaging system	SCD is associated with neurodegeneration in both gray and white matter. Cortical thinning in frontal, temporal, and insular regions, as well as increased white matter mean diffusivity, contributes to cognitive complaints.
				BRDS		
				FAQ		
Na et al. (2024)	Longitudinal observational study	SDC improvement memory function group: 80 mean age age 70.53 ± 6.18	South Korea (Eastern)	SVLT	3.0 T MRI	74.8% of patients with SCD showed memory improvement over 24 months, linked to lower amyloid burden, fewer cardiovascular risks, and stronger executive function. Larger superior parietal lobe volumes were also associated with cognitive improvement.
				SNSB		
				RCFT		
				K-ECog		
				PHQ-9		
	Prospective study			BEPSI-K		
		SDC no				
				PSQI		
				K-HHIE		
		improvement memory function group: 27 mean age 70.96 ± 5.95				
Li et al. (2022)	Cross-sectional observational study with application of machine learning methods	HC: 78 mean age 67.44 ± 6.41	China, Shanghai (Eastern)	MMSE	3.0 T MRI	In addition to the importance of the hippocampus and amygdala, the fimbria, basal nucleus, and cortical nucleus subregions play a significant role, in predicting MMSE and MoCA scores, as well as their changes over time.
				MoCA		
		SCD group: 112 mean age 70.07 ± 7.58				
		MCI group: 36 mean age 74.50 ± 7.67				
Ren et al. (2022)	Cross-sectional study	HC Total Sample: 49 mean age 68.24 ± 5.54	China, Shangai (Eastern)	MMSE	3 T	People with SCD showed preserved hippocampal and parahippocampal volumes, with language and executive function, rather than memory, linked to these regions, highlighting broader cognitive changes in early SCD
				MoCA-B		
				HAMD		
				HAMA		
				IDS-SR		
		SCD Total Sample group: 95 mean age 66.76 ± 4.69				
		HC SubSample: 36 mean age 67.97 ± 5.69				
		SCD SubSample group: 67 mean age 66.64 ± 4.42				
Xu et al. (2020)	Longitudinal case–control design	HC: 20 mean age 71.8 ± 2.9	China, Shanghai, (Eastern)	MMSE	3.0-T MR	ML methods applied to functional connections and topological metrics enables the identification of specific brain connectivity features for diagnosing SCD.
				MoCA		
				AVLT		
				WAIS		
				GDS		
		SCD group: 22 mean age 74.0 ± 5.6		SAS		
				SCD-9		

SCD, Subjective cognitive decline; SCI, subjective cognitive impairment; MCI, Mild cognitive impairment; pSCD, Mild cognitive impairment or dementia; Those who remained stable (sSCD); HC, Healthy control; pDMN, Posterior default mode network; CU, Cognitively Unimpaired; JSRL, Joint neuroimage Synthesis and Representation Learning; MMSE, Mini Mental State Examination; GDS-15, Geriatric Depression Scale; TMT-A, Trail Making Test A; CAMCOG-R, Cambridge Cognitive Assessment-Revised; CVLT, California Verbal Learning Test; BNT, Boston naming test; QSMC, Questionnaire for Subjective Memory Complaints; IADL, Instrumental Activities of Daily Living; MoCA, Montreal Cognitive Assessment; AVLT, Auditory Verbal Learning Test; AVLT-I, AVLT-immediate recall; AVLT-D, AVLT-delayed recall; AVLT-R, AVLT-recognition; CDR, Clinical Dementia Rating; HAMD, Hamilton Depression Rating Scale; ADL, Activities of Daily Living; SCD-I, Subjective Cognitive Decline Initiative; CDR, Clinical Dementia Rating; GDepS, Geriatric Depression Scale; GDS, Global Deterioration Scale; AFT, Animal Fluency Test; BNT, 30-item Boston Naming Test; STT-A and STT-B, Trail Making Test; CDT, Clock Drawing Task; HIS, Hachinski Ischemic Score; CAMCOG, Cambridge Cognitive Examination; WMS, Wechsler Memory Scale; STROOP; SNSB, Seoul Neuropsychological Screening Battery; MFQ, Memory functioning questionnaire; CVLT-II, California Verbal Learning Test-II; MADRS, Montgomery-Asberg depression rating scale; STAI-Trait, Spielberger state–trait anxiety inventory; BRDS, Blessed Rating Dementia Scale; FAQ, Functional Activity Questionnaire; SNSB, Seoul Neuropsychological Screening Battery; RCFT, Rey Complex Figure Test; K-ECOG, Korean Version of the Everyday Cognition Scale; PHQ-9, Patient Health Questionnaire-9; BEPSI-K, Brief Encounter Psychosocial Instrument; PSQI, Pittsburgh Sleep Quality Index; K-HHIE, Korean version of the Hearing Handicap Inventory for the Elderly; SVLT, Seoul Verbal Learning Test; WAIS, Wechsler Intelligence Scale; SAS, Self-Rating Anxiety Scale; SCD-9, Subjective Cognitive Decline Self-administered Questionnaire; MoCA-B, Montreal Cognitive Assessment Basic; HAMD, Hamilton Depression Rating Scale; HAMA, Hamilton Anxiety Rating Scale; IDS-SR, Inventory of Depressive Symptomatology - Self Report; dALFF, Dynamics of Amplitude of Low-Frequency Fluctuations; dReHo, Dynamics of Regional Homogeneity.

#### 3.2.1 Cortical thinning and gray matter volume reductions

Multiple studies have shown that SCD is associated with significant cortical thinning and reductions in gray matter volume (GMV). For example, Cedres et al. (2021) found cortical thinning in the frontal and temporal regions, along with increased mean diffusivity (MD) in white matter. They concluded that gray matter degeneration contributes more to cognitive complaints than white matter degeneration. Similarly, the cross-sectional study by Rivas-Fernández et al. (2023) reported GMV reductions in the frontal gyrus, cingulate cortex, and medial temporal lobe (MTL), as well as white matter reductions in areas such as the inferior frontal gyrus. Furthermore, another study, using several MRI scans on a 3-Tesla scanner, identified hippocampal subfield atrophy in both SCD and AD, with specific involvement of the cornu ammonis (CA1) and subiculum subfields, suggesting that hippocampal atrophy begins early in SCD (Perrotin et al., 2015). Yang et al. (2023) reported left-sided atrophy as a distinguishing feature in MCI, while SCD showed more subtle asymmetry. Atrophy in memory-related regions such as the entorhinal cortex and parahippocampal gyrus, noted by Perrotin et al. (2015) and Lim et al. (2019), supports the idea that these structural changes might signal a transition from SCD to AD. These studies collectively highlight that structural changes, particularly in gray matter and the hippocampus, serve as key early markers of cognitive decline in SCD.

Importantly, evidence from structural MRI and functional imaging studies converge to suggest that early neurodegenerative processes in SCD affect both brain morphology and network dynamics. For example, hippocampal and parahippocampal atrophy—frequently observed in structural studies—is often accompanied by reduced functional connectivity between these same regions and higher-order control networks such as the Default Mode Network (DMN). These complementary findings support a model in which structural deterioration may underlie or parallel connectivity disruptions, providing a multidimensional perspective on preclinical cognitive decline (e.g., Cedres et al., 2021; Liang et al., 2020).

#### 3.2.2 Functional connectivity alterations

Functional connectivity (FC) changes in the brain are also widely reported across studies. Liang et al. (2020) found that a small sample of SCD subjects had reduced resting-state functional connectivity (rsFC) between the hippocampus and regions associated with memory, such as the medial prefrontal cortex (mPFC) and temporoparietal junction (TPJ). These disruptions were correlated with lower cognitive performance. Similarly, Sharma et al. (2021) observed altered connectivity between the posterior Default Mode Network (DMN) and the medial temporal lobe (MTL), which was linked to subjective memory complaints. In contrast, Tian et al. (2024) found increased positive resting-state functional connectivity (RSFC) in the sub-regions of the insula, particularly with executive control network (ECN) and sensorimotor network (SMN) in SCD patients compared to HCs, suggesting early compensatory mechanisms may be at play. In line with these findings, the study by Fu et al. (2022) explored age-related differences in the topological organization of the human connectome using structural MRI data to assess individuals with SCD. The research revealed that SCD participants displayed lower global and local network efficiency, clustering coefficients, and other network properties compared to HCs. These disruptions were more pronounced with aging, particularly impacting the prefrontal lobe and paralimbic system. The findings suggest that SCD individuals have increased vulnerability to cognitive decline, with the topological changes potentially serving as early markers for future cognitive impairment. These studies collectively emphasize that functional connectivity changes, particularly involving memory-related and executive networks, are important markers of SCD and its progression toward AD.

#### 3.2.3 Dynamic functional connectivity and stability

Yang et al. (2021) found that SCD individuals exhibited lower dynamics in memory-related brain regions, such as the hippocampus and parahippocampal gyrus, compared to MCI patients. This suggests more stable, yet potentially inefficient, brain activity in SCD individuals. Wang et al. (2022) further explored dynamic FC states, showing that alterations in sensorimotor and cognitive control networks play a role in early cognitive decline. These findings highlight the importance of examining not only static but also dynamic connectivity changes in SCD, as they may reveal early compensatory mechanisms or the onset of dysfunction.

#### 3.2.4 Advances in network analyses and technological innovations

Graph theory-based approaches and cutting-edge technological methods have emerged as powerful tools for examining brain network properties and identifying early biomarkers of AD progression. Xu et al. (2020) and Ren et al. (2022) both employed graph theory for analyzing functional brain network properties. Xu et al.’s (2020) work highlighted reduced global efficiency and increased modularity, which suggests decreased network integration and increased local segregation in SCD. Ren et al.’s (2022) study, utilizing the MST method, showed preserved global network properties but identified subclinical cognitive changes associated with the hippocampus and parahippocampal regions. The focus on prefrontal and subcortical areas in Xu’s findings complements Ren’s emphasis on hippocampal structures, indicating potential early markers for AD progression.

In parallel, advancements in neuroimaging and ML approaches are enhancing diagnostic precision. van Rooden et al. (2014) used 7-Tesla MRI to study cortical phase shifts, finding significant phase changes in AD patients indicative of iron accumulation. While SCD individuals did not exhibit AD-like phase shifts, increased cortical phase shifts were associated with poorer memory performance, suggesting subtle early changes before cognitive symptoms manifest. The study by Liu et al. (2022) introduced the Joint neuroimage Synthesis and Representation Learning (JSRL) framework which utilizes incomplete multi-modal neuroimaging data to predict SCD conversion. This framework, trained on the Alzheimer’s Disease Neuroimaging Initiative (ADNI) dataset and validated on Chinese Longitudinal Aging Study (CLAS) and Australian Imaging, Biomarkers and Lifestyle (AIBL) datasets, demonstrated improved accuracy and sensitivity in predicting SCD progression. It also emphasized the importance of integrating positron emission tomography (PET) and MRI data for early diagnosis. Similarly, Xu et al. (2020) incorporated ML methods, such as the Least Absolute Shrinkage and Selection Operator (LASSO) and Multiple Kernel-Support Vector Machine (MK-SVM), achieving high diagnostic performance (83.33% accuracy and 90.00% sensitivity) by leveraging graph metrics in resting-state fMRI data.

#### 3.2.5 Cognitive performance and depression

While structural and functional changes are evident in SCD, the relationship between these changes and cognitive performance is more nuanced. Many studies, such as Rivas-Fernández et al. (2023) and Lim et al. (2019), found that SCD patients generally perform similarly to HCs on standard neuropsychological tests, with subtle deficits emerging only in specific tasks, such as semantic verbal fluency. However, Lim et al. (2019) also highlighted that depression scores were higher in patients with progressive SCD, suggesting that mood disorders may be linked to cognitive complaints and disease progression. This connection between cognitive decline and mood underscores the need to assess both cognitive and emotional factors in SCD patients. Li et al. (2022) proposed a ML framework combining sparse coding and random forest (RF) to identify imaging biomarkers for assessing and predicting cognitive function changes in individuals with MCI, SCD, and HCs using MRI. In addition to the hippocampus and amygdala, they found that the fimbria, basal nucleus, and cortical nucleus subregions were particularly important for predicting scores in neuropsychological tests. Further, a prospective study (Na et al., 2024) found that SCD patients with memory improvement exhibited a lower prevalence of amyloid PET positivity and larger volumes in the superior parietal lobes, along with fewer cardiovascular risk factors. These findings suggest that reduced amyloid burden and fewer cardiovascular risks are associated with cognitive improvements, providing potential indicators for clinicians to predict future memory outcomes in SCD.

### 3.3 Risk of bias

The Risk Of Bias In Non-randomized Studies - of Exposures (ROBINS-E) tool was used to assess the risk of bias of the articles included in this review. Figure 2 show the summary of the risk of bias assessment. Out of the total studies assessed, all studies except four (Sharma et al., 2021; Xu et al., 2020; Li et al., 2022; Na et al., 2024) showed low risk of bias due to confounding and one report no information (Fu et al., 2022). Moreover, eight studies (Cedres et al., 2021; Yang et al., 2023; Liang et al., 2020; Sharma et al., 2021; Tian et al., 2024; Liu et al., 2022; Li et al., 2022; Na et al., 2024) show some concerns about bias arising from measurement of the exposure (Szimasky). Further, all studies expect four exhibited some concerns in the selection of participants into the study (Cedres et al., 2021; Yang et al., 2023; Liang et al., 2020; Tian et al., 2024). Seven studies showed some concerns of bias due to post-exposure interventions (Yang et al., 2023; Lim et al., 2019; Liang et al., 2020; Fu et al., 2022; Wang et al., 2022).

Seven studies reported some concerns about the bias due to missing data (Yang et al., 2023; Lim et al., 2019; Liang et al., 2020; Fu et al., 2022; Wang et al., 2022; Ren et al., 2022; Liu et al., 2022). Further, only five studies selected reported some concerns of bias arising from the measurement of the outcome (Tian et al., 2024; Fu et al., 2022; Ren et al., 2022; Liu et al., 2022; Na et al., 2024). Additionally, some studies selected reported some concerns in the selection of the reported result except eight that showed low risk (Rivas-Fernández et al., 2023; Yang et al., 2023; Lim et al., 2019; Tian et al., 2024; Fu et al., 2022; Yang et al., 2021; Xu et al., 2020; Liu et al., 2022; Li et al., 2022).

## 4 Discussion

This review offers a comprehensive analysis of the brain changes associated with SCD, underscoring its potential as a critical preclinical stage for understanding the early markers of AD. The findings highlight the utility of SCD as a model for studying cognitive decline, with a focus on identifying structural and functional changes that may serve as early biomarkers for AD progression.

A central finding from the included studies is the involvement of memory-related brain regions, particularly the hippocampus and medial temporal lobe (MTL), in the early stages of SCD. Structural changes such as hippocampal atrophy and reductions in gray matter volume (GMV) in the MTL were consistently observed across studies (Rivas-Fernández et al., 2023; Cedres et al., 2021). These findings align with earlier research (Bradley, 2002; de Flores et al., 2015) that established the hippocampus as one of the earliest structures affected in AD pathology. The positive correlation between hippocampal GMV and cognitive performance, particularly in delayed recall tasks such as the Auditory Verbal Learning Test (AVLT), emphasizes the sensitivity of episodic memory as an early indicator of cognitive decline (Chen and Mobley, 2019; Dubois et al., 2007).

Moreover, findings from functional MRI studies complement these structural observations by revealing disruptions in brain networks involving these same regions. Specifically, reduced hippocampal and medial temporal lobe volumes, as observed in sMRI studies [e.g., (Rivas-Fernández et al., 2023; Cedres et al., 2021; Perrotin et al., 2015)], are often accompanied by altered connectivity within the DMN and between the hippocampus and medial prefrontal cortex (mPFC) [e.g., (Liang et al., 2020; Sharma et al., 2021)]. These combined patterns suggest that structural degeneration and functional disconnection may co-occur in SCD, reflecting a dual process of anatomical decline and impaired network dynamics. This supports the idea that multimodal imaging offers a more comprehensive view of early neurodegenerative changes than either modality alone. For instance, the study by Liang et al. (2020) identified both structural and functional hippocampal alterations in SCD participants, with structural reductions in gray matter volume paralleling decreased resting-state connectivity between the hippocampus and medial prefrontal cortex. This co-occurrence further supports the link between regional atrophy and disrupted functional communication in memory networks.

Interestingly, studies like Cedres et al. (2021) extended these observations by highlighting cortical thinning in frontal and superior temporal regions, suggesting that cognitive complaints in SCD may not be limited to traditionally AD-vulnerable areas like the parietal cortex. This finding challenges the conventional focus on temporal and parietal regions, suggesting a more heterogeneous neural basis for SCD-related cognitive complaints.

Functional changes in large-scale brain networks were also a prominent theme. Disruptions in the DMN and ECN were observed in both SCD and AD populations, with abnormalities in these networks correlating with memory complaints and early cognitive dysfunction (Sha et al., 2018; Yan et al., 2019). These findings support earlier work (Greicius et al., 2004; Buckner et al., 2008) that linked DMN disruptions to AD progression. Moreover, compensatory mechanisms, as described in studies like Tian et al. (2024), reveal increased connectivity in the SMN and ECN in SCD, suggesting an adaptive response to early cognitive challenges. This aligns with prior research on early-stage AD, which identified similar patterns of compensatory connectivity (Park and Reuter-Lorenz, 2009).

However, the nature of these changes remains debated. While some studies report increased connectivity as a compensatory mechanism, others, such as Xu et al. (2020) and Yang et al. (2021), observed decreased connectivity and reduced network efficiency, pointing to early dysfunction. This duality raises questions about whether these changes represent an attempt by the brain to adapt or the beginning of network failure. Support for the compensatory hypothesis comes from findings of increased connectivity between the left anterior insula and the cerebellum in individuals with SCD, suggesting that specific brain regions may adapt to meet cognitive challenges. These compensatory mechanisms, observed in both the ECN and SMN, may provide a buffer that delays noticeable cognitive decline despite structural and functional changes. Together, these findings help explain why individuals with SCD often maintain relatively preserved cognitive function even as the brain undergoes subtle alterations.

Dynamic functional connectivity (dFC) analyses add further complexity. Yang et al. (2021) and Wang et al. (2022) highlighted reduced connectivity dynamics in memory-related regions and cognitive control networks, suggesting inefficiencies in brain function even before overt cognitive decline. These discrepancies may reflect methodological differences, such as static vs. dynamic FC analyses, or individual variability in how compensatory and degenerative processes coexist in SCD.

Despite these shared findings, some studies report deviations that point to the complexity and heterogeneity of SCD. For example, Griffa et al. (2017) and Vipin et al. (2018) questioned the use of static FC analysis and the number of clusters in analyzing brain networks, suggesting that overly complex models could obscure important connectivity states. Additionally, the inconsistency between studies on the progression of SCD to AD highlights the need for further research. While some studies suggest a clear trajectory from SCD to AD, others, such as van Rooden et al. (2014), did not observe significant changes in cortical phase shifts in SCD, emphasizing the variability in how SCD manifests in different individuals.

An emerging unifying explanation for the resting-state fMRI findings observed in SCD is the disruption of spontaneous low-frequency oscillations (LFOs), which provide the physiological foundation for large-scale functional connectivity. Altered patterns in dynamic connectivity states (Wang et al., 2022) and reduced hippocampal and insular connectivity (Liang et al., 2020; Tian et al., 2024) may reflect impaired coordination of these intrinsic slow oscillations. According to recent evidence (Gong and Zuo, 2025), diminished flexibility and variability in LFOs could impair the brain’s ability to dynamically reconfigure networks, an essential property of cognitive adaptability. This perspective may help reconcile the heterogeneous findings observed across studies and motivate future research on oscillatory biomarkers in early neurodegenerative conditions.

Another emerging area of interest is the involvement of less-studied brain regions, such as the thalamus and hypothalamus. Yang et al. (2021) reported volume reductions in these subcortical structures in SCD, suggesting their potential role in early neurodegenerative processes. This contrasts with earlier studies that predominantly focused on cortical and hippocampal atrophy (Striepens et al., 2010; Cherbuin et al., 2015). These findings underscore the importance of expanding research to include underexplored regions, which may reveal novel biomarkers and pathways of neurodegeneration.

The integration of advanced methodologies, such as graph theory and ML, offers new perspectives on the neural changes associated with SCD. Graph theory analyses by Xu et al. (2020) and Ren et al. (2022) revealed reduced global efficiency and increased modularity in brain networks, indicative of early disruptions in network integration. These findings complement traditional neuroimaging analyses and provide a more nuanced understanding of how SCD affects brain connectivity. Moreover, ML frameworks, such as those developed by Liu et al. (2022), demonstrate the potential for predicting SCD progression by integrating multimodal imaging data. These tools hold promise for improving diagnostic accuracy and identifying subtle brain changes that precede clinical symptoms.

In addition to structural and functional changes, the role of emotional and psychological factors in SCD is increasingly recognized. Growing evidence suggests that depressive symptoms contribute to objective cognitive decline in older adults with SCD, particularly affecting episodic memory and executive function—two cognitive domains highly vulnerable to AD pathology (Diniz et al., 2013; Park et al., 2018; Rapp et al., 2005). Higher depression scores have been consistently associated with progressive SCD, suggesting that mood disorders may interact with or exacerbate cognitive complaints (Lim et al., 2019). Furthermore, depressive symptoms have been linked to biological markers of AD, including increased amyloid-beta burden and reduced hippocampal volume, reinforcing the hypothesis that depression may not only be a psychological response to cognitive concerns but also play a role in accelerating neurodegeneration (Ezzati et al., 2013; Yasuno et al., 2016).

(31)This highlights the need for a holistic approach to SCD research and clinical management, addressing both cognitive and emotional dimensions to fully understand and mitigate disease progression.

### 4.1 Limitations and future directions

The studies included in this review present several limitations that warrant consideration. A significant challenge is the variability in neuroimaging methods across studies, which introduces inconsistencies in data acquisition, analysis, and interpretation. The included studies varied significantly in their methodological approaches. While some studies utilized structural MRI to assess cortical thinning and gray matter volume changes, others employed functional connectivity analyses, resting-state fMRI, or dynamic connectivity assessments. Additionally, sample sizes ranged from small-scale exploratory studies (*N* < 30) to larger, more robust datasets. These variations in study design, population characteristics, and analytical techniques limit the direct comparability of findings and highlight the need for standardized neuroimaging protocols in future research. These methodological differences make direct comparisons between findings difficult and hinder the establishment of unified conclusions about SCD. Moreover, many studies relied on brief neuropsychological assessments to evaluate subjective memory complaints, often neglecting critical factors such as age, education level, and cultural background. These omissions limit the accuracy of assessments and reduce the generalizability of findings to diverse populations (Yang et al., 2023; Liu et al., 2022). Another notable limitation is the insufficient consideration of comorbid conditions, such as mood disorders and other neurodegenerative diseases, which can confound results and obscure the specific neural correlates of SCD (Tian et al., 2024). Mood disorders, in particular, have been shown to influence cognitive complaints, underscoring the importance of addressing emotional and psychological factors in studies of SCD. Furthermore, the predominance of cross-sectional designs and small sample sizes (Yang et al., 2023; Fu et al., 2022; Xu et al., 2020) restricts the ability to infer causal relationships and track the progression of SCD over time. While cross-sectional studies provide valuable insights into brain changes associated with SCD, they do not allow for the assessment of individual trajectories over time. This limitation makes it difficult to distinguish between stable SCD cases and those progressing to MCI or AD. Without longitudinal follow-up, it remains unclear which neurobiological and cognitive markers are truly predictive of disease progression versus those reflecting transient or compensatory changes. To overcome these limitations, future research must prioritize well-designed longitudinal studies with larger and more representative samples. Longitudinal designs are essential for tracking the natural history of SCD, identifying early predictors of progression, and refining risk stratification models. By monitoring individuals over time, researchers can pinpoint the critical transition phases from SCD to MCI and AD, allowing for earlier and more targeted interventions. Furthermore, longitudinal studies will help clarify the dynamic interplay between structural, functional, and molecular changes in the brain, providing a more comprehensive understanding of SCD within the AD continuum. Additionally, harmonizing neuroimaging protocols across studies would improve comparability and facilitate the identification of robust neural correlates. A key contribution of this review is its emphasis on SCD as a heterogeneous condition that occupies a unique position within the AD continuum. The findings suggest that SCD reflects a mix of compensatory mechanisms and early degenerative changes, influenced by individual factors such as age, genetics, and lifestyle. This perspective aligns with cognitive reserve models (Stern, 2002), which propose that variability in brain resilience plays a significant role.

## 5 Conclusion

Overall, this review provides evidence supporting the role of SCD as an early marker of cognitive decline and a valuable model for studying the transition to AD. While findings on structural and functional changes align with much of the existing literature, novel contributions, such as the involvement of less-studied regions and dynamic connectivity changes, highlight the complexity of SCD and its heterogeneous nature. Discrepancies in findings underscore the need for standardized, multimodal, and longitudinal approaches that account for individual variability and comorbidities. By integrating advanced methodologies such as ML and graph theory, future research can uncover novel biomarkers and develop personalized interventions, ultimately advancing our understanding of SCD and its role in neurodegenerative diseases.

## Data Availability

The original contributions presented in the study are included in the article/supplementary material, further inquiries can be directed to the corresponding author.
